# Effect of yoghurt containing *Bifidobacterium lactis *Bb12^® ^on faecal excretion of secretory immunoglobulin A and human beta-defensin 2 in healthy adult volunteers

**DOI:** 10.1186/1475-2891-10-138

**Published:** 2011-12-23

**Authors:** Jayakanthan Kabeerdoss, R Shobana Devi, R Regina Mary, D Prabhavathi, R Vidya, John Mechenro, NV Mahendri, Srinivasan Pugazhendhi, Balakrishnan S Ramakrishna

**Affiliations:** 1 Department of Gastrointestinal Sciences & Dietary Services, Christian Medical College, Vellore & Department of Zoology, Auxilium College, Vellore, India

**Keywords:** Probiotics, innate immunity, health promotion, mucosal defences

## Abstract

**Background:**

Probiotics are used to provide health benefits. The present study tested the effect of a probiotic yoghurt on faecal output of beta-defensin and immunoglobulin A in a group of young healthy women eating a defined diet.

**Findings:**

26 women aged 18-21 (median 19) years residing in a hostel were given 200 ml normal yoghurt every day for a week, followed by probiotic yoghurt containing *Bifidobacterium lactis *Bb12^® ^(10^9 ^in 200 ml) for three weeks, followed again by normal yoghurt for four weeks. Stool samples were collected at 0, 4 and 8 weeks and assayed for immunoglobulin A and human beta-defensin-2 by ELISA. All participants tolerated both normal and probiotic yoghurt well. Human beta-defensin-2 levels in faeces were not altered during the course of the study. On the other hand, compared to the basal sample, faecal IgA increased during probiotic feeding (P = 0.0184) and returned to normal after cessation of probiotic yoghurt intake.

**Conclusions:**

*Bifidobacterium lactis *Bb12^® ^increased secretory IgA output in faeces. This property may explain the ability of probiotics to prevent gastrointestinal and lower respiratory tract infections.

## Introduction

Probiotic foods are widely used to promote health. They are also sometimes used to prevent or treat specific gastrointestinal illnesses. Recent studies have shown that ingestion of foods containing probiotic *Lactobacillus *or *Bifidobacterium *strains prevent or reduce morbidity from enteric infections and lower respiratory tract infections [1,2]. *Bifidobacterium lactis *strain Bb12^® ^is a probiotic microbe that is widely consumed in the form of probiotic yoghurt. Probiotic yoghurt containing this microbe is reported to have beneficial effects on metabolism including lowered serum LDL-cholesterol in patients with type 2 diabetes,[3] increased HDL cholesterol in adult women [4] and improved glucose tolerance during pregnancy [5,6]. Bb12^® ^administration has also been shown to increase faecal secretory IgA excretion in preterm infants [7]. The health claims of probiotics have been demonstrated with varying levels of evidence, with only a few being substantiated using double blind randomized controlled trials.

Dietary practices in India are different from that in the developed countries where evidence of probiotic efficacy has been gathered. This, together with the occurrence of frequent gastrointestinal infections in childhood and the widespread consumption of home-made yoghurt in the diet, may result in differences in the gastrointestinal response to probiotic bacteria in Indians. The present study evaluated the effect of daily ingestion of yoghurt containing *Bifidobacterium lactis *Bb12^® ^on faecal excretion of IgA and β-defensin 2 in healthy adult southern Indian women volunteers.

## Methods

### Participants and Interventions

Healthy young adult women living in a hostel and eating food prepared in the hostel kitchen were recruited for the study. Individuals who had received a course of antibiotics within the last month were excluded as were those who intended to travel out of the city during the course of the feeding trial. Participants were briefed about the nature and purpose of the study, the importance of compliance with the study intervention, and the importance of maintaining a daily record of bowel movements and any abdominal symptoms. Volunteers were given a small monetary incentive to participate in the study. All participants received normal yoghurt daily for the first week of the feeding study, following which they received probiotic yoghurt daily for the next three weeks. This was again followed by regular yoghurt feeding for the next four weeks. Normal yoghurt was prepared in the diet kitchen by boiling standardized toned milk (3.0% fat & 8.5% msnf) and then cooling to 40°C, following which starter culture (YCX-11, Chr Hansen) was added at 1 unit per 10 litres of milk. The milk was distributed in 200 ml cups which were incubated at 40°C until the pH reached 4.6, and cups then transferred to a refrigerator for cooling. Probiotic yoghurt was prepared by adding Bb-12^® ^(Batch no 2927446, Chr Hansen) at a concentration of 0.0006% to the cultured milk prepared as above. This dosage was calculated to provide approximately 10^9 ^cfu of *Bifidobacterium *per 200 ml serving of yoghurt. The investigator responsible for providing the diluted starter culture did not participate in yoghurt distribution or in the laboratory analyses. Bifidobacterial concentrations in yoghurt were checked by culture of diluted yoghurt (1:10 in peptone water broth, 0.9% NaCl, 0.85% peptone) and serial dilutions (10^-2 ^to 10^-10^) were made and plated on Reinforced Clostridial Agar (13.5 g/250 ml, pH 6.8) (Himedia laboratories, Mumbai, India, Catalog number-M154-500G) containing mupirocin (25 μg/L of medium) (RM-6090, Himedia laboratories, Mumbai, India). Plates were incubated at 37°C in anaerobic jars for three days and colony counts calculated from the growth in serial dilutions.

Yoghurt was prepared fresh every morning and distributed at lunch time to the participants. As all participants had lunch in the hostel mess, this allowed distribution at a single point and consumption of yoghurt under supervision. The study was preceded by focus group discussions. Participants were interviewed by a social worker and a dietician. Demographic data were recorded and socioeconomic score was calculated [8]. A 24 hour dietary recall, together with a food frequency questionnaire of commonly used foods over the past three months, was used to calculate nutrient intake using standard tables of the composition and nutritive value of Indian foods [9]. Standard cups and spoons were used to measure meal sizes. Each participant was given a printed study diary and asked to record on a daily basis the following - stool frequency, stool consistency, abdominal pain, gaseousness, and any other symptoms that the participant noted during this period. Each participant was requested to give a fresh sample of stool before the study commenced, at the end of the first intervention period (i.e. at 4 weeks) and at the end of the second intervention period (i.e. at 8 weeks). The study protocol, incentive, and consent forms were approved by the Institutional Review Board. All participants provided written consent.

Fresh samples of stool were collected in the morning and transported immediately to the laboratory and stored at -80°C. Secretory IgA and beta-defensin 2 were assayed by ELISA (K8870 and K6500, Immunodiagnostik, Germany) following manufacturer's instructions. Stool samples weighing 80-120 mg were diluted with appropriate amounts of buffer provided in the kit to give constant dilutions, vortexed, and centrifuged at 13000 rpm for 5 minutes in 1.5 ml tubes. Supernatants were diluted 1:250 in wash buffer. Standards, controls and stool samples were simultaneously run in the ELISA and the concentration of secretory IgA and β-defensin 2 were read against a standard curve and expressed as concentration per gram wet weight of stool.

### Statistics

Values are presented as numbers for categorical variables and as median with interquartile range for continuous data. Analysis of significance of differences between groups was done using paired t tests comparing each group with the others.

## Results

26 women of median age 19 (range 18-21) years were recruited for the study. Their median (range) socioeconomic score was 16 (13-25). A score of 11-15 is categorized as middle class and 16-25 as upper middle class). Their median (range) height was 154 (150-171) cm, weight was 53.5 (43-77) kg, and BMI was 22.1. (18.5-26.3). The dietary intakes and preferences were as follows. Eight were lacto-vegetarian, while the rest ate meat at least once a week. Their median (range) daily nutrient intake was energy 1425 (1025-1735) kcal, protein 30 (20-40) g, fat 19 (16-26) g, simple carbohydrates 17 (14-45) and complex carbohydrates 246 (143-336).

No significant change in bowel frequency (median daily frequency 1) or stool consistency was reported during consumption of the probiotic yoghurt. There was no significant complaint of abdominal pain or bloating or other abdominal symptoms during probiotic yoghurt feeding compared to normal yoghurt feeding. Two volunteers each had abdominal pain during probiotic yoghurt feeding and normal yoghurt feeding. 13 and 8 participants respectively had symptoms consistent with a common cold during three weeks of probiotic or normal yoghurt feeding.

**Figure 1:**
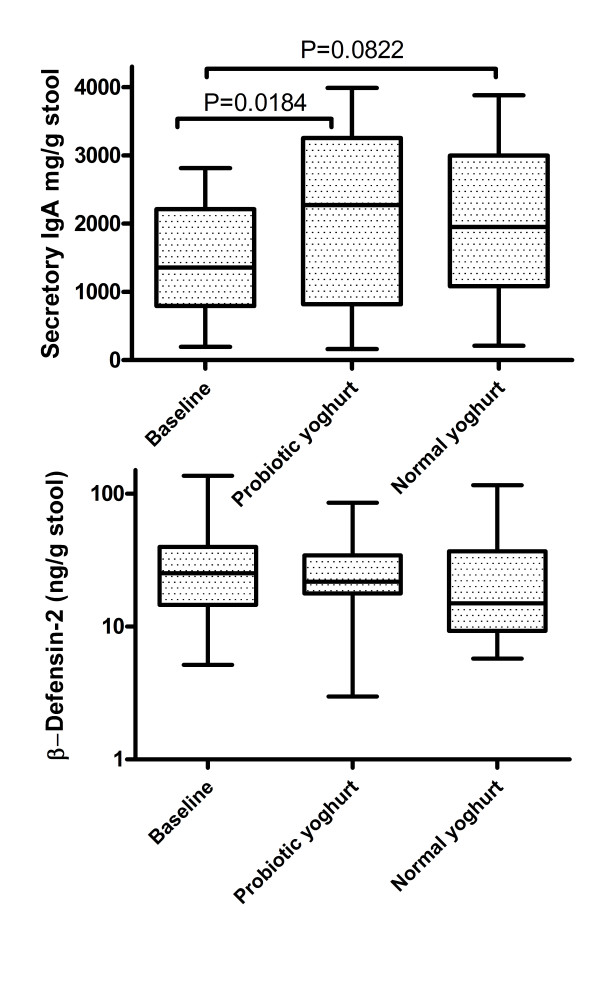
**Box and whiskers plots showing the effect of probiotic yoghurt containing *Bifidobacterium lactis *Bb12^® ^on faecal excretion of secretory IgA and human beta-defensin-2**. The middle line represents the median, the box represents the interquartile range and the whiskers represent the total range. Faecal IgA excretion significantly increased during after probiotic yoghurt feeding and returned to normal levels after cessation of the probiotic.

Figure 1 shows the β-defensin 2 concentrations in faeces of volunteers fed probiotic yoghurt or normal yoghurt. There was no change in the concentration of this immune effector molecule brought about by the ingestion of probiotics. On the other hand, faecal levels of secretory IgA were significantly higher in the participants during the probiotic yoghurt feeding period compared to basal periods (P = 0.0184). Faecal IgA levels after cessation of probiotic yoghurt feeding showed a trend towards being increased compared to the baseline levels (P = 0.0822) and was not significantly different from the peak levels observed during probiotic yoghurt feeding (P = NS) (Figure 1).

## Discussion

This study in a group of young adult women in south India shows that the probiotic yoghurt was well tolerated and that faecal excretion of secretory immunoglobulin A was significantly increased during feeding of probiotic yoghurt compared to the baseline. Although the levels decreased after probiotic yoghurt was stopped, they did not return to normal and tended to be higher than baseline levels. This suggests that the effect of the probiotic yoghurt persisted even after cessation of feeding this; there is also be a possibility that normal yoghurt contributed to elevated faecal sIgA, that was more pronounced during feeding of probiotic yoghurt. These results are likely to be generalizable. Studies in healthy children and preterm infants have shown an increase in faecal IgA excretion in response to probiotic interventions [7,10]. A study in animals showed that probiotic yoghurt containing Lactobacillus casei increased IgA secretion in mice [11]. On the other hand, a study in infants did not find any change in faecal IgA after probiotic administration [12]. In another study, faecal IgA output in pre-term infants was increased in response to both mother's milk and to fermented formula (in which the probiotic was killed by heating) [13].

Probiotics are also known to upregulate defensin expression and to increase defensin secretion from intestinal epithelium. Both lactobacilli and probiotic *E. coli *have been shown to increase human beta-defensin 2 (hBD-2) secretion from CaCo-2 cells through stimulation of TLR2 and TLR5 respectively [14-16]. In a feeding trial in healthy adult volunteers, the probiotic *E. coli *Nissle strain increased fecal hBD-2 excretion in healthy adult volunteers [17]. In the present study, there was no obvious effect on faecal hBD-2 excretion during the probiotic yoghurt feeding period. Thus far, an effect on human beta-defensin expression has been noted only with lactobacilli and with E. coli Nissle 2. In a study where 11 lactobacilli were tested, only 2 of the strains increased hBD-2 mRNA expression in CaCo-2 cells. The difference between lactobacilli strains in their ability to induce hBD-2 correlated with the presence of genes encoding glycosylated cell surface structures [18]. Although we did not examine the probiotic used in this study for these genes, this appears a likely explanation for the absence of an effect on hBD-2 excretion in stool.

This study had several drawbacks. It was a non-randomised study and the participants could potentially differentiate between the two different kinds of yoghurt on the basis of smell and taste, although the nature of the two was not known to them. These were not commercially available standardized products, although we took care to make them to a standard protocol. The generalizability of these results to other products and populations is therefore not to be assumed.

The present study appears to be the first report of an effect of probiotic foods on faecal IgA in healthy adults. Increased IgA output was noted only with yoghurt containing Bb12^® ^and not with normal yoghurt. An increase in faecal IgA may explain the ability of probiotics to prevent diarrhoeal illness in specific settings. It is possible that this may also explain beneficial effects of probiotics in preventing severe lower respiratory tract infections.

## Competing interests

The study was supported by a grant from Chr. Hansen (India) Private Limited. The funder participated in the design of the study, provided the starter cultures and probiotics, and was involved in initial training of the dieticians in manufacturing and quality control of the yoghurt. The funder was not involved in laboratory analyses, data analysis, data reporting, or write-up of the manuscript.

## Authors' contributions

JK and PD were responsible for specimen processing and laboratory analyses; RSD was responsible for dietary analyses; RSD, NVM and SP were responsible for yoghurt preparation and quality control; RMR, RV and JM were responsible for recruitment and supervision of the feeding study; SP was responsible for overall supervision; BSR, RMR, JK and SP were responsible for design; JK and BSR were responsible for write-up of the manuscript. All authors read and approved the final manuscript.
